# High Levels of Antibiotic Resistance Patterns in Two Referral Hospitals during the Post-Ebola Era in Free-Town, Sierra Leone: 2017–2019

**DOI:** 10.3390/tropicalmed6020103

**Published:** 2021-06-16

**Authors:** Zikan Koroma, Francis Moses, Alexandre Delamou, Katrina Hann, Engy Ali, Freddy Eric Kitutu, Juliet Sanyu Namugambe, Doris Harding, Veerle Hermans, Kudakwashe Takarinda, Pruthu Thekkur, Isatta Wurie

**Affiliations:** 1 Ministry of Health and Sanitation, Freetown P.O. Box 232, Sierra Leone; franqoline@gmail.com (F.M.); dorisharding@yahoo.com (D.H.); 2 Department of Chemical Pathology, College of Medicine and Allied Health Sciences, University of Sierra Leone, Freetown P.O. Box 232, Sierra Leone; imwurie@aol.com; 3 Maferinyah Training and Research Centre, Forécariah 4099, Guinea; adelamou@maferinyah.org or; 4 Africa Centre of Excellence (CEA-PCMT), University Gamal Abdel Nasser, Conakry 1017, Guinea; 5 Sustainable Health Systems, Freetown P.O. Box 232, Sierra Leone; hann.katrina@gmail.com; 6 Luxembourg Operational Research Unit (LuxOR), Medical Department, Medecins Sans Frontieres—Operational Centre Brussels, L-1617 Luxembourg, Luxembourg; engy.ali@luxembourg.msf.org (E.A.); Veerle.HERMANS@luxembourg.msf.org (V.H.); 7 Strengthening Pharmaceutical Systems (SPS), Department of Pharmacy, Makerere University School of Health Sciences, Kampala P.O. Box 7072, Uganda; kitutufred@gmail.com; 8 Department of Pharmacy, Mbarara University of Science and Technology (MUST), Mbarara P.O. Box 1410, Uganda; julietsanyu@gmail.com; 9 Centre for Operational Research, International Union Against Tuberculosis & Lung Disease (The Union), 75006 Paris, France; ktakarinda@theunion.org (K.T.); pruthu.tk@theunion.org (P.T.)

**Keywords:** AMR, laboratory, AMR surveillance, antimicrobial stewardship, operational research, SORT IT, Sierra Leone

## Abstract

The Post-Ebola era (2017–2019) presented an opportunity for laboratory investments in Sierra Leone. US CDC supported the Ministry of Health and Sanitation to establish a microbiological unit for routine antimicrobial sensitivity testing in two referral (pediatric and maternity) hospitals in Freetown. This study describes resistance patterns among patients’ laboratory samples from 2017 to 2019 using routine data. Samples included urine, stool, cerebrospinal fluid, pus-wound, pleural fluid, and high vaginal swabs. Selected Gram-positive and Gram-negative bacterial isolates were tested for antimicrobial susceptibility. Of 200 samples received by the laboratory, 89 returned positive bacterial isolates with urine and pus-wound swabs accounting for 75% of positive isolates. The number of positive isolates increased annually from one in 2017 to 42 in 2018 and 46 in 2019. Resistance of the cultures to at least one antibiotic was high (91%), and even higher in the pediatric hospital (94%). Resistance was highest with penicillin (81%) for Gram-positive bacteria and lowest with nitrofurantoin (13%). Gram-negative bacteria were most resistant to ampicillin, gentamycin, streptomycin, tetracycline, cephalothin and penicillin (100%) and least resistant to novobiocin (0%). Antibiotic resistance for commonly prescribed antibiotics was high in two referral hospitals, highlighting the urgent need for antimicrobial stewardship and access to reserve antibiotics.

## 1. Introduction

The estimated 700,000 deaths occurring globally every year due to antimicrobial resistance (AMR) have elevated its global importance to among the top three health and development threats facing humanity [1]. In the African region, there is a paucity of established AMR surveillance systems as highlighted in a 2014 WHO report [1,2]. Information about the status of AMR in West Africa is limited [3,4] although one systematic review in 2017 showed that AMR was common in that region particularly in hospitalized patients with frequent use of first-line antibiotics. And yet, there is weak diagnostic capacity and limited availability of second-line treatment options [5].

For Sierra Leone in particular, there is only limited data on AMR. One study conducted during the 2014–2016 West African Ebola outbreak using laboratory samples (urine and fomite swabs) in an outpatient department in a hospital in Bo district (Sierra Leone), revealed that nearly 86% of the isolates were multiple drug resistant (MDR) [6]. In addition, this study showed that 64% of the isolates produced an extended-spectrum ß-lactamase (ESBL) resistance pattern [6]. During the same outbreak, most Ebola virus disease (EVD) patients (suspected or confirmed) were routinely given Ciprofloxacin and Cefixime to cover gut bacterial translocation [7]. Concerns were raised regarding the possible over prescription of antibiotics as a routine hospital and community-based treatment and the practice of self- and non-clinician prescriptions on a wider scale even after the outbreak [6].

To date, there is no established AMR surveillance system in Sierra Leone, although plans are on course to establish one [6]. Therefore, identifying clinical isolates and resistance patterns at the health facility level could inform antimicrobial stewardship interventions and contribute to combating antibiotic resistance [8,9]. To remedy the situation and as part of the Post-Ebola Health System Recovery and Strengthening Plan, between 2017 and 2018, the Centers for Disease Control and Prevention (CDC), Atlanta, supported the Ministry of Health and Sanitation (MoHS) to establish a microbiology unit in the laboratory serving two referral hospitals, the pediatric Ola During Children’s Hospital (ODCH) and maternal Princess Christian Maternity Hospital (PCMH) in Freetown. This microbiology unit was equipped to perform full bacteriology analysis—culture and antibiotic sensitivity testing (AST). This pilot intervention enhanced routine microbiology services for clinicians and provided information on appropriate antimicrobial therapy to support AMR surveillance in both hospitals and facilitate tracking of AMR patterns. The intervention also focused on capacity building for antimicrobial sensitivity testing. This development provided an opportunity to assess the extent of bacteriological resistance to antibiotics and contribute to better surveillance in Sierra Leone. The results of this study and lessons learnt are crucial to inform the Ministry of Health and Sanitation for not only patient management but for future hospital-based AMR surveillance and antimicrobial stewardship.

This study assessed the presence of bacterial isolates, antimicrobial susceptibility and factors associated with AMR among routinely collected laboratory samples between 2017 and 2019 in two referral hospitals in Freetown, Sierra Leone.

## 2. Materials and Methods

### 2.1. Study Design

This was a descriptive study that analyzed routine hospital laboratory data.

### 2.2. Study Settings (General and Specific)

The study was conducted in Ola During Children’s Hospital (ODCH) and Princess Christian Maternity Hospital (PCMH) in Freetown. Both hospitals are the only tertiary hospitals for referrals for pediatric and maternity patients in the capital city. They are situated in the same compound and share a common laboratory which hosts the microbiology unit. Further, samples from these two facilities were taken to the common laboratory hosting the microbiology units for culture and sensitivity testing.

ODCH and PCMH receive average monthly inpatient admissions of 1000 and 850, respectively. Their common laboratory provides service for hematology, biochemistry and microbiology including GeneXpert for tuberculosis. The average monthly number of samples analyzed by the laboratory was 1870.

Among other routine tests, the microbiology unit performs antimicrobial susceptibility testing and bacterial identification. Clinical samples cultured included urine, blood, stool, cerebrospinal fluid (CSF), pus from wounds, pleural fluid, and high vaginal swabs (HVS). Standard operating procedures for culture techniques are employed for all the clinical samples received.

The antimicrobial susceptibility tests were performed using the Kirby-Bauer disk diffusion technique, where standardized inoculum (0.5 McFarland) from a pure culture was seeded onto sterile Mueller Hinton Agar [10]. Excess moisture on the agar surface was allowed to be absorbed before application of the antimicrobial disks. Gram-positive and Gram-negative antibiotic disks were selected for their bacterial isolates. The plates were incubated aerobically at 35 ± 2 °C for 18–24 h, after which zones of inhibition were measured using calipers and interpretation done using the Clinical and Laboratory Standards Institute (CLSI) 2017 Performance Standards for Antimicrobial Susceptibility Testing [10]. Based on the CLSI guidelines, inhibition zones were reported as Sensitive, Intermediate or Resistant. The disks and their concentrations in micrograms included: penicillin G (PEN: 1), ampicillin (AMP: 10), nalidixic acid (NAL: 30), tetracycline (TET: 30), nitrofurantoin (NIT: 50), gentamicin (GEN: 10), ciprofloxacin (CIP: 5), erythromycin (ERY: 5), cephalothin (CEP: 30), kanamycin (KAN: 30), streptomycin (STR: 10), trimethoprim/sulfamethoxazole (SXT: 1.2), chloramphenicol (CHL:30) and colistin (COL: 25). These selected antibiotics are commonly prescribed to treat bacterial infections in the general population [11,12].

Bacteria were identified using the microscopic technique. The biochemical tests were performed to identify the Gram-negative isolates, including lactose fermentation, urease, indole and triple sugar iron, and the tube catalase and coagulase to identify the different species of Gram-positive isolates.

### 2.3. Study Population and Period

The study population included all bacterial samples from the pediatric and maternity hospitals with positive isolate cultures undergoing AST, from 2017 to 2019.

### 2.4. Data Collection Source

All samples received for AST at the microbiology unit of ODCH-PCMH laboratory were entered manually into a report ledger and a Microsoft Excel database daily. The results were updated following confirmatory testing and completion of AST verification. Variables recorded included age, sex, reasons for investigation, type of sample, pathogens isolated, and antibiotics used for sensitivity testing. A second laboratory lead double-checked the laboratory database, and a final verification was made by the consultant medical laboratory scientist. The national consultant further checked all information to verify the results and hence conduct a random 10% check on the ledger and request forms for compliance.

### 2.5. Data Analysis and Statistics

The laboratory electronic database (in Microsoft Excel, Microsoft, Redmond, WA, USA) was imported into Stata 16 software (StataCorp, College Station, TX, USA) for analysis. A descriptive analysis was performed to determine frequencies and proportions from categorical variables. Pearson’s chi-square test was used to compare proportions of positive isolates for selected variables with a level of significance set at *p* < 0.05 and 95% confidence level.

## 3. Results

### 3.1. Demographic and Clinical Characteristics

Of the 200 requests received from both ODCH and PCMH, 89 samples returned positive bacterial isolates, of which 26 (29%) were from the maternity hospital and 63 (71%) from the pediatric hospital. Thirty-three positive isolates (37%) were from males whilst 56 (65%) were from females. By age, four (5%) were neonates, 27 (30%) were infants, 30 (34%) were children and 27 (30%) were adults.

From the two referral hospitals, the number of positive bacterial isolates cultured for antibiotic sensitivity testing increased annually from one in 2017, to 42 in 2018 to 46 in 2019. Urine and pus-wound swabs accounted for 75% of all positive isolates. More than half (59%) of the cultured bacterial isolates were from skin and urogenital origin (Table 1).

### 3.2. Quarterly Trend of Lab Samples That Showed Bacterial Growth

**Figure 1:**
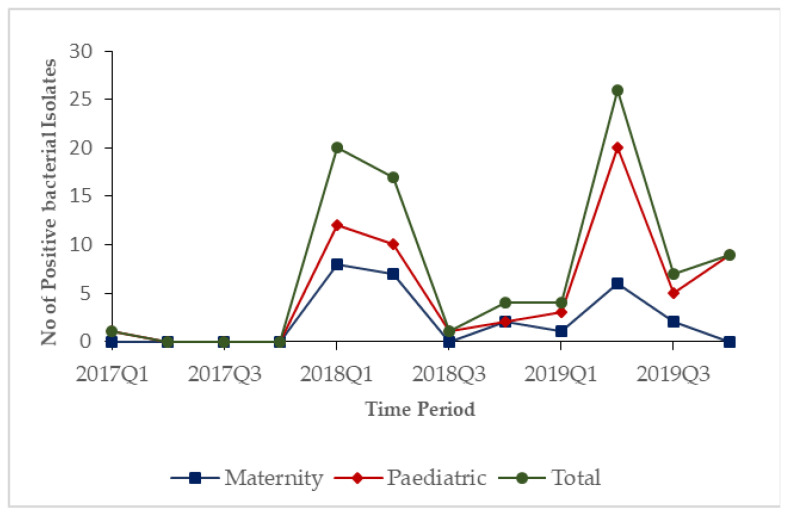
Quarterly trends of lab samples that showed bacterial growth stratified by hospitals in Freetown, Sierra Leone, from 2017–2019.

In 2017, only one positive bacterial isolate was observed. During 2018 and 2019, the numbers of positive bacterial isolates started increasing in the first quarters and peaked in the second quarters of each year before dipping in the third and fourth quarters in both hospitals (Figure 1).

### 3.3. Antimicrobial Resistance Patterns

Antimicrobial resistance to at least one antibiotic was observed among the 81 (91%) cultures with positive bacterial growth. There was a higher proportion of resistance in the pediatric hospital’s samples (94%) compared to those of the maternity hospital (85%). The proportion of resistance was higher among neonates (100%), and among males (97%). Among specimen types, the proportion of resistance ranged from 83% for pleural fluid to 100% for CSF and stool. The proportion of resistance among Gram-positive and Gram-negative bacteria was almost identical (approximately 90%) (Table 2).

### 3.4. Factors Associated with Antimicrobial Resistance

There were no significant associations between antimicrobial resistance among cultures with bacterial growth and factors investigated: hospital type, time period, patient age and gender, specimen type, organ system, laboratory department or Gram-staining (all *p*-values > 0.05) (Table 2).

### 3.5. Antibiotic Sensitivity Testing

Table 3 presents the antibiotic sensitivity testing and antimicrobial resistance patterns stratified by Gram-staining among samples with positive bacterial isolates. Overall, 52% of the samples tested were resistant. Antibiotic resistance was significantly higher among Gam-negative bacteria (59%) than Gram-positive bacteria (46%), *p* = 0.0448. Among Gram-positive bacteria, the proportion of antibiotic resistance was highest with penicillin (81%) and kanamycin (71.4%), and lowest with nitrofurantoin (13%). Among Gram-negative bacteria, antibiotic resistance was highest with six antibiotics: ampicillin, gentamycin, streptomycin, tetracycline, cephalothin and penicillin (100%) and lowest with novobiocin (0%) (Table 3).

## 4. Discussion

This study is one of a limited number of studies reporting on laboratory surveillance of antimicrobial resistance in Sierra Leone in the aftermath of the 2014/2016 EVD outbreak. We found that culture and sensitivity testing during the study period remained low. Most samples with positive bacterial isolates were from the pediatric hospital and among female patients. The finding of this study is similar to studies done in Ethiopia and Nigeria respectively, where there were high positive bacterial isolates found among female patients [13,14]. However, our study found more female positive bacterial isolates from both pediatric and maternity hospitals while in Ethiopia and Nigeria, higher positive bacterial isolate were only reported from pediatric samples.

Antimicrobial resistance was higher in the pediatric hospital, among neonates, males, and in CSF and stool specimens. Similarly, despite using higher sample size, two studies from Nigeria and Egypt found higher positive bacterial isolate among the male neonates [15,16]. There were no statistically significant associations with AMR for factors assessed in this study. Similarly, Youssef et al., in Egypt, found no statistically significant associations with factors assessed in their study exploring the epidemiology of urinary tract infection among neonates in a single intensive care unit [16].

Our findings indicate a low utilization of the laboratory during the study period in terms of culture and sensitivity testing despite a relative increase over time. This might be related to the important gaps in documentation of laboratory requests and samples received for culture and sensitivity testing we found. Further, because the unit was newly established, one reason might be the lack of awareness from health care workers about the availability of the services. Further, as culture and sensitivity testing were provided for free, some Health Care workers might chose to refer their requests to partner external laboratories which grant them percentages of examination costs. In addition, samples selected for testing may have been those from patients who were not showing clinical improvements after empirical antibiotic treatment and hence, were a subset of potential cultures. The laboratory also experienced challenges with a shortage of trained technicians, lack of clinical outcome data, frequent stockouts of supplies and electricity outages, all of which reduced its performance. Another study by Lakoh et al., in Sierra Leone, also highlighted an urgent need for investment in microbiological diagnostics infrastructure and antimicrobial stewardship across the country which contrast with the low use of the available capacities in our study context [17]. This calls for better awareness raising among users but also with hospitals staff to increase the use and documentation of services.

There are important public health implications from our study. First, there is a needs to improve and increase the use of the existing laboratory capacity for culture and drug sensitivity testing not only for better patient management but also as part of surveillance for antimicrobial resistance and improved antibiotic stewardship in the study hospitals and in the country. One approach would be to use the laboratory as referral structure for more health facilities in the area where it is established to increase demand for culture. Second, the laboratory could be integrated in the national AMR surveillance network, which would provide venue for continuous capacity building and provision of supplies. For this to happen, better support from the Ministry of Health and Sanitation in terms of infrastructure, capacity building, human resources, equipment and reagents is needed.

Finally, the low use of available services may be explained by the lack or insufficient trained laboratory technicians, frequent stockouts in supplies or the frequent electricity shortages at the laboratory. Whatever the reasons are, the situation is concerning, especially due to the high levels of AMR found for antibiotics listed by WHO as critically (cephalosporins 3rd to 5th generation and quinolones) and highly (cephalosporins 1st to 2nd generation and penicillin) important human use [18]. Therefore, strong leadership from the study hospitals and the MoH are needed to improve demand for services and inform AMR surveillance strategies in Sierra Leone.

## 5. Conclusions

The study highlighted a low uptake of antibiotic sensitivity testing by clinicians in the two referral hospitals in Sierra Leone, and high resistance to commonly prescribed antibiotics in those hospitals. This was in spite of a newly established, dedicated laboratory with this capacity. There is a need for urgent action to improve the use of AST to drive antimicrobial stewardship in these hospitals, and to expand the spectrum of available antibiotics to include ‘reserve’ antibiotics in clinical care.

## Figures and Tables

**Table 1:** Demographic and clinical characteristics of patients with positive bacterial isolates on cultured AST from two referral hospitals (pediatric and maternity) in Freetown, Sierra Leone from 2017 to 2019.

Characteristic	Pediatric		Maternity		Total	
	*n*	(%) *	*n*	(%) *	*n*	(%) *
Total	63	(100.0)	26	(100.0)	89	(100.0)
Age						
Neonates (0–30 days)	4	(6.4)	0	(0.0)	4	(4.5)
Infants (1 month–2 years)	27	(42.9)	0	(0.0)	27	(30.3)
Children (2–18 years)	29	(46.0)	1	(3.9)	30	(33.7)
Adults (>18 years)	2	(3.2)	25	(96.1)	27	(30.3)
Gender						
Male	32	(50.8)	1	(3.9)	33	(37.1)
Female	31	(49.2)	25	(96.1)	56	(62.9)
Year						
2017	1	(1.6)	0	(0.0)	1	(1.1)
2018	25	(39.7)	17	(65.4)	42	(47.2)
2019	37	(58.7)	9	(34.6)	46	(51.7)
Specimen types						
CSF	5	(7.9)	1	(3.9)	6	(6.7)
HVS	0	(0.0)	9	(34.6)	9	(10.1)
Pleural Fluid	6	(9.5)	0	(0.0)	6	(6.7)
Pus-wound swabs	25	(39.7)	4	(15.4)	29	(32.6)
Stool samples	0	(0.0)	1	(3.9)	1	(1.1)
Urine samples	27	(42.9)	11	(42.3)	38	(42.7)
Organ system involved						
Respiratory	7	(11.1)	0	(0.0)	7	(7.9)
Skin	19	(30.2)	3	(11.5)	22	(24.7)
Urogenital	24	(38.1)	6	(23.1)	30	(33.7)
Others	7	(11.1)	16	(61.5)	23	(25.8)
Not recorded	6	(9.5)	1	(3.9)	7	(7.9)
Lab Department						
Microbiology	62	(98.4)	26	(100.0)	88	(98.9)
Haematology	1	(1.6)	0	(0.0)	1	(1.1)

* Column percentage; CSF = Cerebrospinal Fluid; HVS = High Vaginal Swab; AST = Antibiotic Sensitivity Testing.

**Table 2:** Factors associated with antimicrobial resistance among cultures with bacterial growth from two referral hospitals in Freetown, Sierra Leone from 2017 to 2019.

Characteristic	Total	Antimicrobial Resistance *		*p*-Value
		*n*	(%)	
Total	89	81	(91.0)	
Hospital type				
Paediatric	63	59	(93.7)	0.175
Maternity	26	22	(84.6)	
Time Period				
2017	1	1	(100.0)	0.104
2018	42	41	(97.6)	
2019	46	39	(84.8)	
Age				
Neonates (0–30 days)	4	4	(100.0)	0.755
Infants (1 month-2 years)	27	25	(92.6)	
Children (2–18 years)	30	28	(93.3)	
Adults (>18 years)	27	23	(85.2)	
Gender				
Male	33	32	(97.0)	0.131
Female	56	49	(87.5)	
Specimen types				
CSF	6	6	(100.0)	0.920
HVS	9	8	(88.9)	
Pleural Fluid	6	5	(83.3)	
Pus-wound swabs	29	27	(93.1)	
Stool samples	1	1	(100.0)	
Urine samples	38	34	(89.5)	
Organ system involved				
Respiratory	7	6	(85.7)	0.394
Skin	22	20	(90.9)	
Genital	30	29	(96.7)	
Urinary	23	19	(82.6)	
Others	7	7	(100.0)	
Lab Department				
Microbiology	88	80	(90.9)	0.752
Haematology	1	1	(100.0)	
Gram staining				
Gram Positive	44	40	(90.9)	0.973
Gram Negative	45	41	(91.1)	

CSF = Cerebrospinal fluid; HVS = High vaginal swab; * Resistance to at least one antibiotic.

**Table 3:** Antibiotic sensitivity testing and antimicrobial resistance patterns stratified by Gram-staining among samples with positive bacterial isolates from two referral hospitals (pediatric and maternity) in Freetown, Sierra Leone, from 2017 to 2019.

Antibiotic	Gram-Positive, *n* = 246				Gram-Negative, *n* = 213				Total, *n* = 459			
	Tested		Resistant		Tested		Resistant		Tested		Resistant	
	*n*		*n*	(%) #	*n*		*n*	(%) #	*n*		*n*	(%) #
Cephalothin	3		2	(66.7)	5		5	(100.0)	8		7	(87.5)
Chloramphenicol	35		9	(25.7)	34		22	(64.7)	69		31	(44.9)
Gentamycin	12		5	(41.7)	1		1	(100.0)	13		6	(46.2)
Ciprofloxacillin	17		6	(35.3)	26		6	(23.1)	43		12	(27.9)
Colistrin Sulphate	5		3	(60.0)	10		4	(40.0)	15		7	(46.7)
Kanamycin	7		5	(71.4)	21		15	(71.4)	28		20	(71.4)
Nalidixic Acid	7		4	(57.1)	27		14	(51.9)	34		18	(52.9)
Nitrofurantoin	8		1	(12.5)	18		8	(44.4)	26		9	(34.6)
Tetracycline	21		10	(47.6)	2		2	(100.0)	23		12	(52.2)
Trimethoprim/ Sulfamethoxazole	29		17	(58.6)	19		17	(89.5)	48		34	(70.8)
Ampicillin	4		1	(25.0)	4		4	(100.0)	8		5	(62.5)
Ceftriaxone	2		1	(50.0)	17		14	(82.4)	19		15	(78.9)
Streptomycin	9		4	(44.4)	3		3	(100.0)	12		7	(58.3)
Erythromycin	34		19	(55.9)	3		2	(66.7)	37		21	(56.8)
Oxacillin	29		10	(34.5)	2		1	(50.0)	31		11	(35.5)
Penicillin	16		13	(81.3)	1		1	(100.0)	17		14	(82.4)
Imipenem	2		1	(50.0)	19		6	(31.6)	21		7	(33.3)
Novobiocin	6		2	(33.3)	1		0	(0.0)	7		2	(28.6)

Percentage calculated with the number of Gram-positive, Gram-negative and total as denominator; # Percentage calculated with the number tested as a denominator.

## Data Availability

The data presented in this study are available on request from the corresponding author.
